# Facile Synthesis of Monodispersed Titanium Nitride Quantum Dots for Harmonic Mode-Locking Generation in an Ultrafast Fiber Laser

**DOI:** 10.3390/nano12132280

**Published:** 2022-07-01

**Authors:** Ya-Tao Yang, Han-Wei Wu, Yuan Zou, Xue-Yang Fang, Shuang Li, Yu-Feng Song, Zhen-Hong Wang, Bin Zhang

**Affiliations:** Institute of Translational Medicine, First Affiliated Hospital (Shenzhen Second People’s Hospital), Health Science Center, College of Electronics and Information Engineering, Shenzhen University, Shenzhen 518060, China; yatao86@szu.edu.cn (Y.-T.Y.); 00910023@pku.edu.cn (H.-W.W.); 2070436032@email.szu.edu.cn (Y.Z.); xueyang0210.fang@connect.polyu.hk (X.-Y.F.); 2070245066@email.szu.edu.cn (S.L.); yfsong@szu.edu.cn (Y.-F.S.)

**Keywords:** titanium nitride (TiN), quantum dots (QDs), saturable absorber (SA), fiber laser, harmonic mode locking

## Abstract

As a member of the transition metal nitride material family, titanium nitride (TiN) quantum dots (QDs) have attracted great attention in optical and electronic fields because of their excellent optoelectronic properties and favorable stability. Herein, TiN QDs were synthesized and served as a saturable absorber (SA) for an ultrafast fiber laser. Due to the strong nonlinear optical absorption characteristics with a modulation depth of ~33%, the typical fundamental mode-locked pulses and harmonics mode-locked pulses can be easily obtained in an ultrafast erbium-doped fiber laser with a TiN-QD SA. In addition, at the maximum pump power, harmonic mode-locked pulses with a repetition rate of ~1 GHz (164th order) and a pulse duration of ~1.45 ps are achieved. As far as we know, the repetition rate is the highest in the ultrafast fiber laser using TiN QDs as an SA. Thus, these experimental results indicate that TiN QDs can be considered a promising material, showing more potential in the category of ultrafast laser and nonlinear optics.

## 1. Introduction

In fiber lasers, ultrafast pulses with a high repetition rate are widely used, such as optical communication [1,2], frequency combs [3,4], and optical sampling [5]. For resonators with a high repetition rate, the single pulse energy and peak power are relatively low, which can effectively avoid some nonlinear effects in the amplification process, thereby improving the overall efficiency of the fiber amplifier [6]. At present, there are mainly two methods in fiber lasers to achieve high-repetition-rate pulses, one is to shorten the cavity length of the fiber laser resonator, and the other is passively harmonic mode-locked technology [7]. Because the repetition rate of a fiber laser is inversely proportional to the cavity length, if the repetition rate reaches the order of 100 MHz, the cavity length based on shortening the cavity length must be limited to less than two meters [8,9]. Each component has its own physical size, and the length of the gain fiber will be limited, resulting in insufficient optical gain. Space optical elements may also be used, which greatly reduce the mobility of fiber lasers [10,11,12]. The most important thing is that with the increase in the repetition frequency in the short cavity, the corresponding energy of a single pulse will decrease, which may not be enough to produce a nonlinear effect, thus leading to the inability of the laser to enter lock mode. Relatively speaking, passively harmonic mode-locking technology [13,14] has more advantages because there is no restriction on the length of the gain medium and cavity, and the repetition rate of the harmonic pulse obtained is much higher than the basic repetition rate. Generally, for the sake of achieving high-order harmonic mode locking, the simplest and most intuitive method is to increase the pump power.

However, the saturable absorber (SA) is an important part of a mode-locked fiber laser resonator. At present, SAs can be divided into two types, one is an equivalent SA, such as nonlinear polarization rotation (NPR) [15,16] and a nonlinear amplification loop mirror (NALM) [12,17,18,19], and the other is a natural SA, such as graphene [20,21,22], carbon nanotubes [23,24], topological insulators (TI) [25,26,27], and semiconductor saturated absorber mirrors (SESAMs) [28,29,30]. Fiber lasers with an NPR structure can be used for achieving ultrashort pulses but are sensitive to polarization and are easily influenced by the environment [31]. However, based on the property, the NPR fiber laser can generate various pulse phenomena, providing an excellent playground for exploring these nonlinear pulse dynamics [32,33,34,35]. Apart from generating the nonlinear pulse phenomena [36], a mode-locked fiber laser based on the NALM technique can be insensitive to the external environment through all polarization maintaining the fiber structure and retaining long-term stable operation [37,38], which makes it a reliable pulse light source and shows more potential applications in many fields, including supercontinuum generation and optical frequency combs [39,40]. However, it is difficult to realize self-starting mode-locking [41]. Compared with equivalent SAs, natural SAs have more advantages. Because the modulation depth of natural SAs is relatively large, there is no need to fine-tune the polarization controller. Generally, the saturation recovery time is shorter, and it is easier to promote the generation of ultrashort pulses [42]. For decades, the materials used for pulse generation as SAs have been gradually discovered by researchers [43]. There are two main types, semiconductor materials typically represented by SESAMs, and nanomaterials such as graphene and carbon nanotubes. Among them, some other nanomaterials with nonlinear optical absorption properties have emerged, such as black phosphorus [44], topological insulators, and layered metal disulfide such as molybdenum disulfide (MoS_2_) [13,45], tungsten disulfide (WS_2_) [46], and tin disulfide (SnS_2_) [47,48], etc. These materials have been widely used as SAs for ultrafast fiber lasers [13,49,50]. Even so, it is still necessary to explore new nanomaterials and to investigate their nonlinear optical absorption properties to meet the application requirements of different fields. Recently, titanium nitride (TiN) quantum dots (QDs), as the components of transition metal nitride materials, show many excellent properties [51,52,53], including a large nonlinear optical response and high environmental stability. Therefore, they are suitable to use as potential SAs for ultrafast photonics [54,55]. Review [56] describes the current mainstream material synthesis technologies and some other proposed technologies for the synthesis of saturable absorbers. Some researchers have also proposed a new method to produce SAs from plastic materials and ethylene glycol [57].

In this work, we prepared fresh TiN QDs through a solvothermal method mentioned in reference [44], and then fabricated the TiN-QD SA on microfiber by using optical deposition technology. The nonlinear optical absorption characteristics of the TiN-QD SA in the communication band were measured by the Z-scan technique. The corresponding modulation depth was about 33%, showing that the TiN-QD SA had good nonlinear optical absorption. Then, the prepared TiN-QD SA was put into the fiber ring cavity, and then the passive mode-locked fiber laser with the TiN-QD SA was built. Based on the saturable absorption characteristics, typical fundamental mode-locked pulses with a central wavelength of 1559 nm, 3 dB bandwidth of 0.39 nm, and repetition rate of 6.15 MHz were realized. At higher input power, the harmonic mode-locking phenomenon was also observed. When increasing the input power from 100 mW to 580 mW, the repetitive frequency changed from 135.43 MHz (22nd order) to 1.009 GHz (164th order). As far as we know, the repetition rate with a ~1 GHz level is the highest one among the ultrafast fiber lasers with TiN-QD SAs.

## 2. Preparation, Characterization, and Nonlinear Optical Properties of Titanium Nitride Quantum Dots

Titanium nitrate quantum dots (TiN QDs) were constructed by modifying a solvothermal method [56] reported in our previous work [58], where in this work we increased the ratio of the glycerol and ammonia treatment program to obtain monodispersed ultrasmall TiN QDs. In brief, 500 mg of tetrabutyl titanate (>99%, GC grade, Macklin Inc., Shanghai, China), 12 mL of glycerol (99%, AR grade, Macklin Inc., Shanghai, China), 20 mL of methanol (99.5%, AR grade, Macklin Inc., Shanghai, China), and 12 mL of ethyl ether (99%, AR grade, Macklin Inc., Shanghai, China) were mixed under vigorous stirring for 1 h, followed by a solvothermal process at 150 °C for 15 h. The resultant products were collected via centrifugation with a centrifuge (Thermal Scientific, SORVALL ST 16R, Waltham, MA, USA) at 12,000 rpm for 15 min and washed with ethanol (99.5%, AR grade, Macklin Inc., Shanghai, China) 3 times, followed by drying in a vacuum oven (Memmert, VO500, Schwabach, Germany) at 70 °C overnight. After that, the product was placed in a program-controlled heating tubular furnace (HF Kejing Inc., Hefei, China), and the precursors were first annealed in ammonia gas flow (mixed with Argon gas, 10 *v*/*v*% for ammonia gas, Air Liquide) at 450 °C for 2 h and were then annealed at 700 °C for another 2 h with a controlled heating process (25 °C to 450 °C, 5 °C/min; 450–700 °C, 3 °C/min).

**Figure 1:**
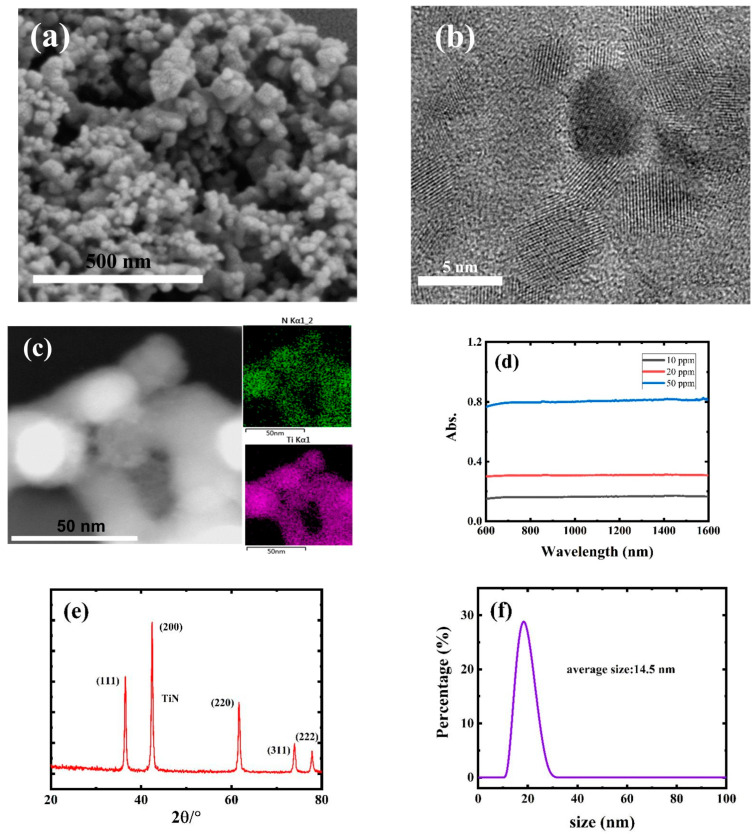
(**a**) SEM image. (**b**) TEM image. (**c**) EDX mapping result. (**d**) Linear optical absorption behavior. (**e**) XRD pattern and (**f**) DLS analysis of TiN QDs.

As shown in Figure 1, SEM (scanning electron microscope, FEI Scios, Thermo Fisher, Waltham, MA, USA) (Figure 1a) and TEM (transmission electron microscope, JEM-F200, JEOL, Tokyo, Japan) (Figure 1b) images proved that the as-synthesized TiN nanomaterials maintained a mono-dispersed size distribution at 10–20 nm, confirming that QDs were successfully fabricated. EDX (energy-dispersive X-ray spectroscopy, JEM-F200, JEOL, Tokyo, Japan) (Figure 1c) demonstrated that Ti and N elements were distributed across the skeleton of the TiN QDs, suggesting a well-configured structure, which was further consolidated by the XRD (X-ray diffraction, MiniFlex600, Tokyo, Japan) pattern in Figure 1e. In addition, Figure 1d shows the linear optical absorption of the TiN QD solution; the absorption value at 1550 nm reached ~0.8 at a concentration of only 50 ppm, demonstrating that there was an exciting absorption at the communication bands. In Figure 1e, the XRD result demonstrated that the characteristic peaks corresponding to (111), (200), (220), (311), and (222) were well preserved with no peaks corresponding to titanium oxide, indicating negligible TiN oxidation and an intact TiN structure. DLS (dynamic light scattering) (Figure 1f) also indicated that the TiN had good dispersion in isopropanol, with a similar size to that observed in SEM and TEM. With small sizes and relatively high surface free energy, QDs have been reported to suffer from stability issues. Experiments were conducted to check whether the obtained TiN QDs could maintain the original structure in solution.

**Figure 2:**
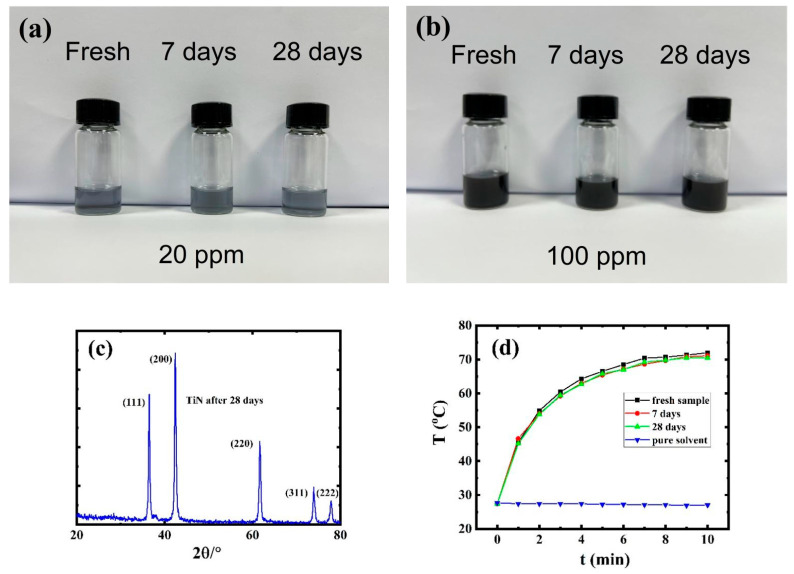
(**a**,**b**) Photographs of TiN QD solutions after different time durations (20 ppm for (**a**) and 100 ppm for (**b**)). (**c**) XRD pattern of the TiN QDs after 28 days and (**d**) photothermal behavior under irradiation with an 808 nm laser of a 50 ppm TiN QD solution after different time durations (laser power: 1 W/cm^2^).

As shown in Figure 2a,b, the isopropanol solution of TiN quantum dots showed almost no color change and no obvious precipitate formation. TiN quantum dots exhibited good stability in isopropanol solution within four weeks. The XRD result (Figure 2c) of the QDs after 28 days unambiguously proved that the TiN QDs did not undergo crystalline change or oxidation. To make the conclusion more convincing, the photothermal behavior of QDs after dispersal in water for a different number of days was tested, as shown in Figure 2d. Extraordinary photothermal efficiency was observed for TiN QDs under irrdiation with an 808 nm laser, and to our satisfactory, this material displayed almost identical performance as the freshly prepared TiN QDs. Such excellent stability of the TiN QDs in both air and solution together with the above-mentioned optical absorption performance makes this material a promising candidate for various applications.

## 3. Experimental Setup

**Figure 3:**
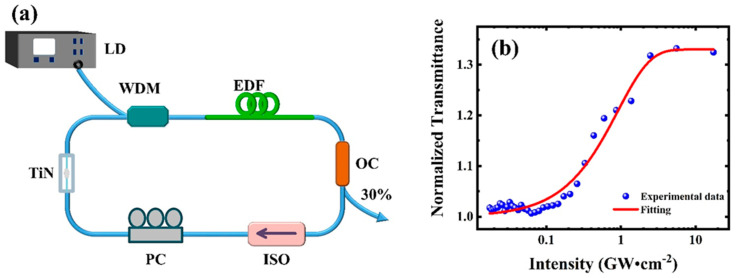
(**a**) Experimental diagram. (**b**) Nonlinear absorption transmission curve of TiN-QD SA.

The TiN QDs were used for the fiber laser. Figure 3a shows the experimental setup. A laser diode with a central wavelength of 980 nm was used as the pump source and was coupled to the resonant cavity by a wavelength division multiplexer (WDM). A 1.2 m erbium-doped fiber (EDF80, OFS, Norcross, GA, USA) was used as the gain medium, and its peak absorbance at 1530 nm was 80 dB/m, and its dispersion at 1550 nm was 61 ps^2^/km. The prepared TiN-QD SA was located between the WDM and polarization controller (PC). The polarization controller can control the polarization state of light in the optical fiber. Finally, the laser passed through a 70:30 output coupler (OC), in which 30% of the laser output was used for monitoring outside the cavity, while the remaining 70% of the laser continued to operate unidirectionally in the cavity through the polarization-independent isolator (PI-ISO). All optical fiber components in the cavity were welded by a welding machine. Except EDF, any optical fibers in the resonant cavity were standard single-mode fibers (SMFs), with a length of about 32.2 m and a dispersion value of −23 ps^2^/km at 1550 nm. Thus, the length of the whole ring resonator was ~33.4 m, and the corresponding net dispersion value was −0.67 ps^2^. The characteristic curves of the output laser pulse were detected by a spectrometer (AQ6370D, Yokogawa, Tokyo, Japan), an oscilloscope (DSOS104A, Keysight, CO, USA, 1 GHz, 20 Gs/s), a radio-frequency spectrometer (N9322C, Keysight, CO, USA), and an autocorrelation instrument (FR-103XL, Femtochrome, Berkeley city, CA, USA). We prepared TiN-QD SA on microfibers by optical deposition technology [59] (see details in the Supplementary Materials; Figures S1 and S2 in the Supplementary Materials show schematic diagram of the fabrication of the TiN-QD SA device on microfiber and the microscopic image of the as-prepared TiN-QD SA, respectively; reference [59] is cited in the Supplementary Materials). As shown in Figure 3b, the characteristics of TiN-QD SA on microfiber deposited by TiN were further studied. The nonlinear optical absorption characteristics [60] of the TiN-QD SA in the communication band were measured by a Z-scan technique [61]. It can be seen that the modulation depth was about 33%, which indicates that the TiN QD material has excellent saturable absorption characteristics and can be used in mode-locked devices of fiber lasers.

## 4. Experimental Results and Discussion

### 4.1. Fundamental Mode Locking

**Figure 4:**
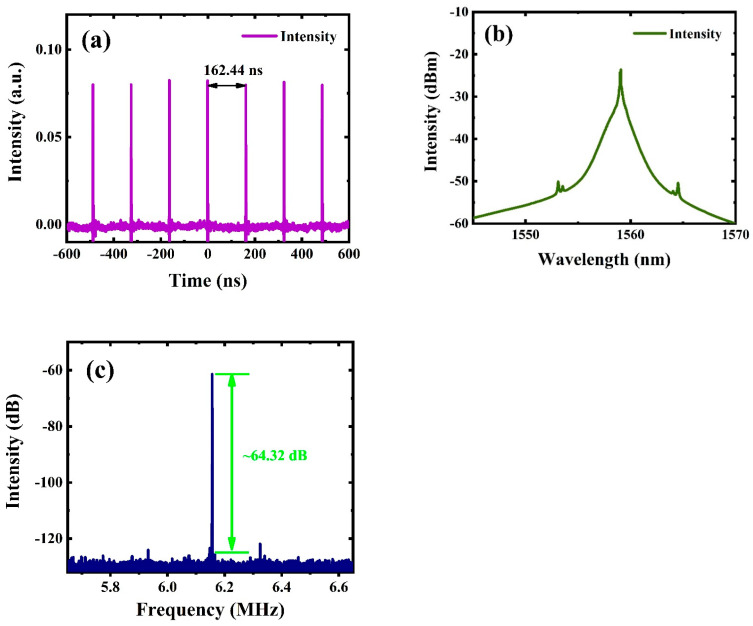
Characteristic diagram of output pulses at 30.4 mW. (**a**) Pulse sequences. (**b**) Optical spectrum. (**c**) RF spectrum.

In this work, through an increase in the pump power and proper modulation of the angles of the PC, the laser realized stable mode-locked pulses at lower input power. When the pump power was 30.4 mW, the output characteristics of the laser can be seen in Figure 4. Figure 4a–c indicates the corresponding temporal trains, optical spectrum, and radio frequency (RF) spectrum, respectively. As can be clearly seen from Figure 4a, the fundamental repetition frequency had a temporal interval of ~162.44 ns, which corresponded to the repetition frequency of ~6.15 MHz, and the cavity length was ~33.4 m. Figure 4b displays the optical spectrum of the fundamental mode-locked pulses. Clearly, a peak appeared at the central wavelength of 1559 nm, and the bandwidth of 3 dB was 0.39 nm. At this time, the low pump power and weak pulse energy reduced the sideband intensity in the spectrum and made it inconspicuous [62]. When the pump power was continuously increased, this phenomenon improved: the sideband became more obvious, and the 3 dB bandwidth increased obviously. Furthermore, there was a high peak at 6.15 MHz in the RF spectrum, and the signal-to-noise ratio (SNR) was ~64.32 dB, which makes it clear that the laser operated in a relatively stable state, as shown in Figure 4c.

### 4.2. Harmonic Mode Locking

**Figure 5:**
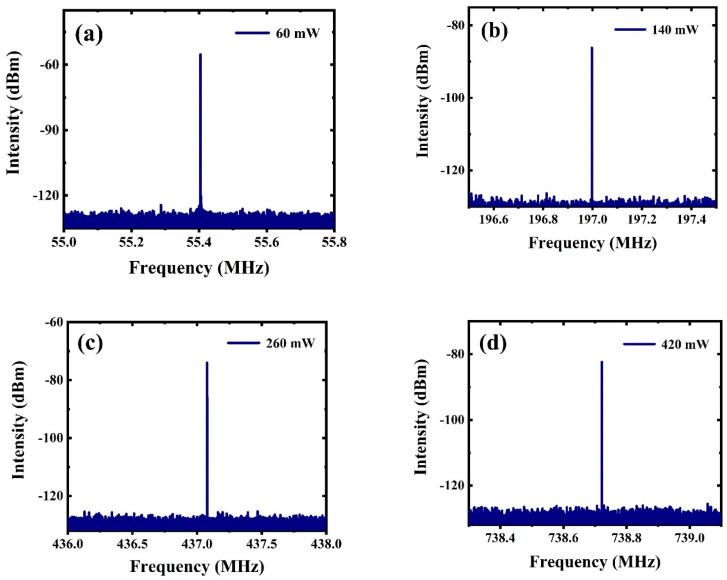
RF spectra of harmonic output pulses at 60 mW (**a**), 140 mW (**b**), 260 mW (**c**), and 420 mW (**d**).

**Figure 6:**
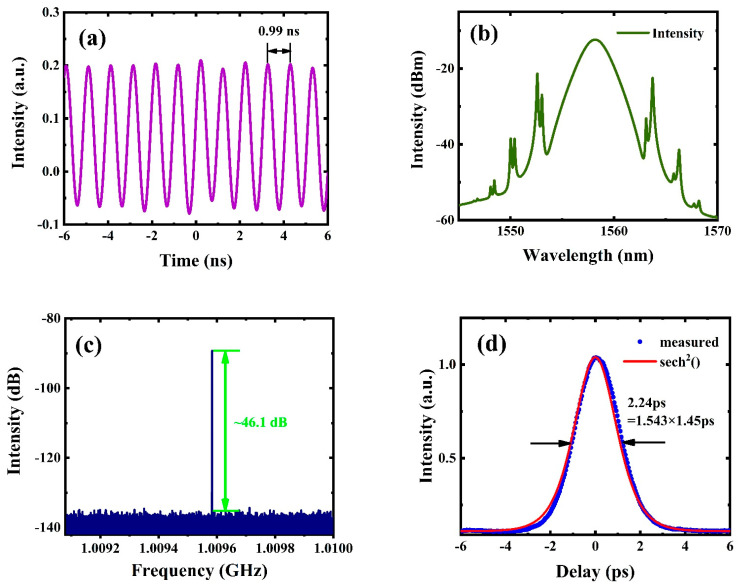
Characteristic diagrams of output pulses at 580 mW. (**a**) Pulse trains. (**b**) Spectrum. (**c**) RF spectrum. (**d**) Autocorrelation curve.

When the pump power increased gradually, stable harmonic mode-locked pulses were generated in the fiber laser by adjusting the PC. Figure 5a–d shows the RF spectra of different harmonic orders at pump powers of 60 mW, 140 mW, 260 mW, and 420 mW, respectively. When the pump power reached 60 mW, the repetition rate was 55.4 MHz, corresponding to the 9th harmonic order. Moreover, the repetition rates at 140 mW, 260 mW, and 420 mW were 197 MHz, 437.1 MHz, and 738.35 MHz, corresponding to the 32nd, 71st, and 120th harmonic orders, respectively. Figure 6 shows the characteristic diagram of the 164th harmonic pulse realized at the pump power of 580 mW. The temporal pulse trains are shown in Figure 6a. According to the figure, the temporal interval between adjacent pulses was ~0.99 ns, and the repetition frequency was about 1.009 GHz. Figure 6b shows the spectrogram in the same state. Clearly, the central wavelength and 3 dB spectral bandwidths were 1559 nm and 2.32 nm, respectively. Obvious Kelly sidebands were observed, which shows that optical solitons were generated in the fiber laser cavity. At this time, in this case, since the pump power level was higher than the fundamental repetition rate, the continuous wave component disappeared. As shown in Figure 6c, a clear high peak of ~1.009 GHz was observed, and the SNR was ~46.1 dB. The autocorrelation curve is presented in Figure 6d. The full width at half maximum (FWHM) was about 2.24 ps, and the pulse width was estimated to be 1.45 ps (assuming that the pulse was fitted by using the Sech^2^ curve). Thus, the calculation shows that the time-bandwidth product (TBP) was about 0.415 (the Sech^2^ pulse conversion limit is 0.315), which indicates that the output pulse was slightly chirped.

**Figure 7:**
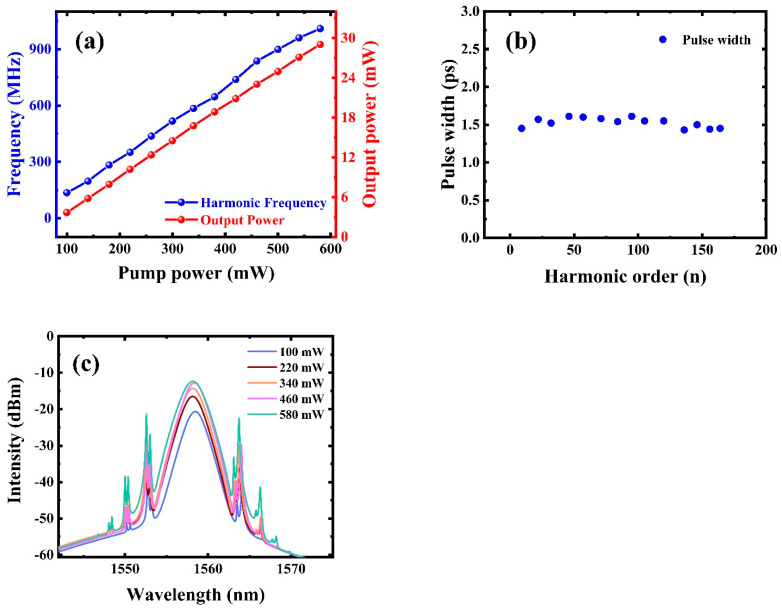
(**a**) Repetition rates and output powers at different pump powers. (**b**) Pulse widths under different orders. (**c**) Optical spectral properties at different pump powers.

Then, the repetition rate and output powers with different pump powers were evaluated. Figure 7a shows the growth relationship between the repetition rates and the output powers as a function of the pump power. The input power increased from 100 mW to about 580 mW, and the pulse repetition rate almost increased linearly from 135.43 MHz (22nd order) to 1.009 GHz (164th order). As seen in this figure, there was a linear relationship between the output power and the pump power. The growth slope was about 5.3%. When the pump power reached the maximum value of 580 mW, the average output power was about 29.04 mW, corresponding to a lower pulse energy. In addition, the pulse widths under different harmonic orders were evaluated, as shown in Figure 7b. With the increment in harmonic order, the pulse width of the harmonic mode-locked pulses remains between 1.43 ps and 1.61 ps. Figure 7c illustrates the optical spectra of the harmonic mode locked under different pump powers. According to the figure, with increasing pump power, the spectral width of the optical spectra gradually widened, and the corresponding intensities also increased, while the central wavelengths of the optical spectra did not change significantly, i.e., retained at around 1559 nm, indicating these harmonic mode-locked pulses were relatively stable. Due to limited experimental conditions, the long-term stability of harmonic pulses in the fiber laser with the TiN-QD SA could not be evaluated. In fact, the above results mainly focused on highlighting the potential applications of TiN-QD material in the nonlinear optics, suggesting that it can be used as a promising nonlinear optical material. Interestingly, by using TiN-QDs as the SA in fiber lasers, ultrashort pulses with a ~1 GHz repetition rate can be obtained. To the best of our knowledge, this is the highest repetition frequency in a mode-locked fiber laser with TiN material as the SA.

## 5. Conclusions

TiN QD material was prepared for the first time and was used to make promising semiconductor lasers in harmonic mode-locked fiber lasers. The characterization parameters and nonlinear optical absorption of the TiN QDs were also evaluated. Because of its excellent nonlinear optical property, the performance of the erbium-doped fiber laser with the TiN-QD SA was demonstrated. Under proper pump power and in a polarization state, the fundamental mode locking and harmonic mode locking can be realized. The output pulses of the fundamental repetition rate had a central wavelength of 1559 nm and a repetitive frequency of 6.15 MHz. Furthermore, an adjustment of the polarization controller and a change in the pump power allowed harmonic mode locking. At the pump power of 580 mW, the repetition rate reached a high level of ~1 GHz, corresponding to the 164th harmonic order. In addition, with increasing pump power, the harmonic order and output power also increased linearly. These results of the fundamental mode locking and harmonic mode locking together show that TiN QDs are potential materials for use as nonlinear devices for pulse lasers. It is believed that this study can promote the rapid development and application of TiN QDs in ultrafast photonics and nonlinear optical devices.

## Data Availability

Not applicable.

## Supplementary Materials

The following supporting information can be downloaded at: https://www.mdpi.com/article/10.3390/nano12132280/s1, Figure S1: Schematic diagram of the fabrication of the TiN SA device on microfiber; Figure S2: Microscopy image of the as-prepared TiN QD SA.
