# Supplementary material for: Facile Synthesis of Monodispersed Titanium Nitride Quantum Dots for Harmonic Mode-Locking Generation in an Ultrafast Fiber Laser
Source: Nanomaterials (Basel). 2022 Jul 1;12(13):2280. doi: 10.3390/nano12132280 (PMC9268693; doi:10.3390/nano12132280)
Supplement: Supplementary file 1 [file nanomaterials-12-02280-s001.zip › nanomaterials-1774032-supplementary.pdf]

nanomaterials

Supporting Information

Facile Synthesis of Monodispersed Titanium Nitride Quantum Dots for Harmonic Mode-Locking Generation in an Ultrafast Fiber Laser

Ya-Tao Yang †, Han-Wei Wu †, Yuan Zou, Xue-Yang Fang, Shuang Li, Yu-Feng Song, Zhen-Hong Wang * and Bin Zhang *

Institute of Translational Medicine, First Affiliated Hospital (Shenzhen Second People's Hospital), Health Science Center, College of Electronics and Information Engineering, Shenzhen University, Shenzhen 518060, China; yatao86@szu.edu.cn (Y.-T.Y.); 00910023@pku.edu.cn (H.-W.W.); 2070436032@email.szu.edu.cn (Y.Z.); xueyang0210.fang@connect.polyu.hk (X.-Y.F.); 2070245066@email.szu.edu.cn (S.L.); yfsong@szu.edu.cn (Y.-F.S.)

* Correspondence: wangzhenhong@szu.edu.cn (Z.-H.W.); bin_zhang13@u.nus.edu (B.Z.)

† These authors contributed equally to this work.

The microfiber-based TiN QDs saturable absorber (SA) is fabricated by using the optical deposition method, which is similar to the previous reports[1]. The experimental setup for the optical deposition technique is shown in Figure S1. Herein, the microfiber draw from the single-mode fibers (SMFs) has a waist diameter of ~ 8 μm. Besides, the microfiber has a low insertion loss of 0.5 dB at communication band. As shown in Figure S1, the output power of the laser source is ~100 mW. First, we dropped the proper amount of TiN QDs solution which covers the waist region of microfiber. Then we turned on the laser source and the optical deposition of TiN QDs started. The deposited amount of TiN QDs is proportional to the deposition time. Thus, in order to control the deposition amount of TiN QDs, the deposition process was in situ observed by a microscope with a magnification of 40-folds. When the deposition amount is appropriate, we turned off the laser source and the deposition process was completed. Finally, the fabricated TiN QDs SA was evaporated at room temperature. The TiN QDs saturable absorber is characterized by using the fluorescent microscope, as shown in Figure S2.

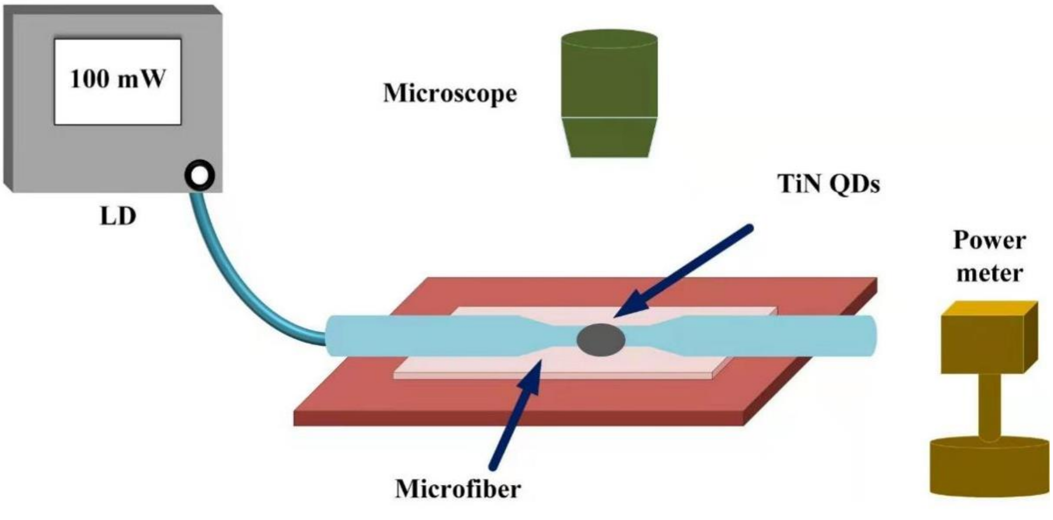

Figure S1. Schematic diagram of the fabrication of TiN QDs SA device on microfiber.

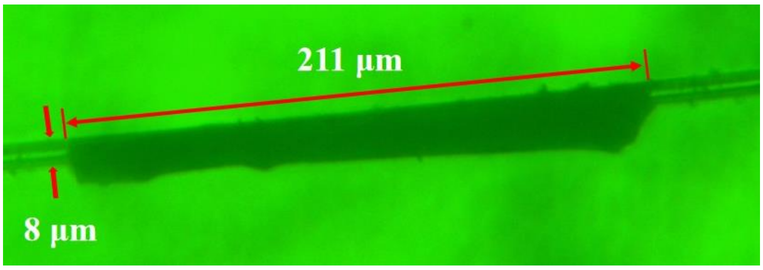

Figure S2. Microscopy image of the as-prepared TiN QDs SA.

References

1. Kashiwagi, K.; Yamashita, S. Deposition of carbon nanotubes around microfiber via evanascent light. *Opt. Express* **2009**, *17*, 18364–18370, doi:10.1364/OE.17.018364.
